# Prevalence and risk factors for proximal deep vein thrombosis at admission in patients with traumatic fractures: a multicenter retrospective study

**DOI:** 10.3389/fcvm.2024.1372268

**Published:** 2024-04-25

**Authors:** Xiaobing Liu, Peng Pang, Zhenguo Luo, Wenbo Cai, Wangyang Li, Jianhong Hao

**Affiliations:** ^1^ Department of Anaesthesiology, HongHui Hospital, Xi'an JiaoTong University, Xi’an, Shaanxi, China; ^2^ Department of Anaesthesiology, Binzhou Medical College Affiliated Hospital, Binzhou, Shandong, China; ^3^ Emergency Department, Linfen Hospital Affiliated to Shanxi Medical University, Linfen, Shanxi, China

**Keywords:** lower extremity fracture, pelvic-acetabular fracture, proximal venous thromboembolism, admission, risk factor

## Abstract

**Objective:**

This study aimed to determine the associated risk factors for proximal deep vein thrombosis (DVT) in patients with lower extremity and pelvic-acetabular fractures.

**Methods:**

The medical records of 4,056 patients with lower extremity and pelvic-acetabular fractures were retrospectively reviewed. The patients were classified into proximal or non-proximal DVT groups. Logistic regression models were used to determine the independent risk variables for proximal DVT. The predictive value of the related risk factors was further analyzed using receiver operating characteristic curves.

**Results:**

The prevalence of proximal DVT was 3.16%. Sex, body mass index (BMI), fracture site, injury mechanism, diabetes, coronary heart disease (CHD), injury-to-admission interval, hematocrit, platelet counts, and D-dimer levels differed significantly between the two groups. BMI ≥ 24.0 kg/m^2^, femoral shaft fractures, high-energy injury, diabetes, injury-to-admission interval >24 h were independent risk factors for proximal DVT. CHD decreased the risk of proximal DVT. The platelet and D-dimer had high negative predictive value for predicting proximal DVT formation, with cut-off values of 174 × 10^9^/L and 2.18 mg/L, respectively.

**Conclusion:**

BMI ≥ 24.0 kg/m^2^, femoral shaft fractures, high-energy injury, diabetes, injury-to-admission interval >24 h were independent risk factors for proximal DVT in patients with lower extremity and pelvic-acetabular fractures. Platelet count and D-dimer level were effective indicators for excluding proximal DVT occurrence. CHD decreased the risk of proximal DVT.

## Introduction

Venous thromboembolism (VTE) is a common complication after traumatic injury (1–3). Pulmonary embolism (PE) is a potentially fatal condition in patients with fractures and can occur within 72 h after a trauma (4). The prevalence of early PE after trauma can be as high as 10%–42% (5). Studies have confirmed that VTE is a major risk factor for PE (6). Therefore, being able to predict deep vein thrombosis (DVT) in patients with traumatic fracture at the time of admission for implementing appropriate interventions is of considerable clinical importance.

Clinically, lower extremity DVT is classified as distal DVT (isolated calf vein thrombosis) or proximal DVT (thrombosis involving the popliteal vein and above) (7). Studies have shown that patients with distal DVT only are less likely to have PE (8, 9). Compared with distal DVT, proximal DVT is considered to be more prone to PE (10). Therefore, exploring and analyzing the prevalence and associated risk factors of proximal DVT at admission in patients with traumatic fractures to achieve early detection, early diagnosis, and early treatment, and prevent PE and death are important.

However, to date, there are no reports on the risk factors of proximal thrombosis at admission in patients with trauma. To address this knowledge gap, we conducted a multicenter retrospective study. In this study, we reviewed the medical records of individuals with lower extremity and pelvic-acetabular fractures admitted to three hospitals in China between February 2018 and March 2023, and analyzed the prevalence and associated risk factors for proximal DVT at admission.

## Materials and methods

**Figure 1:**
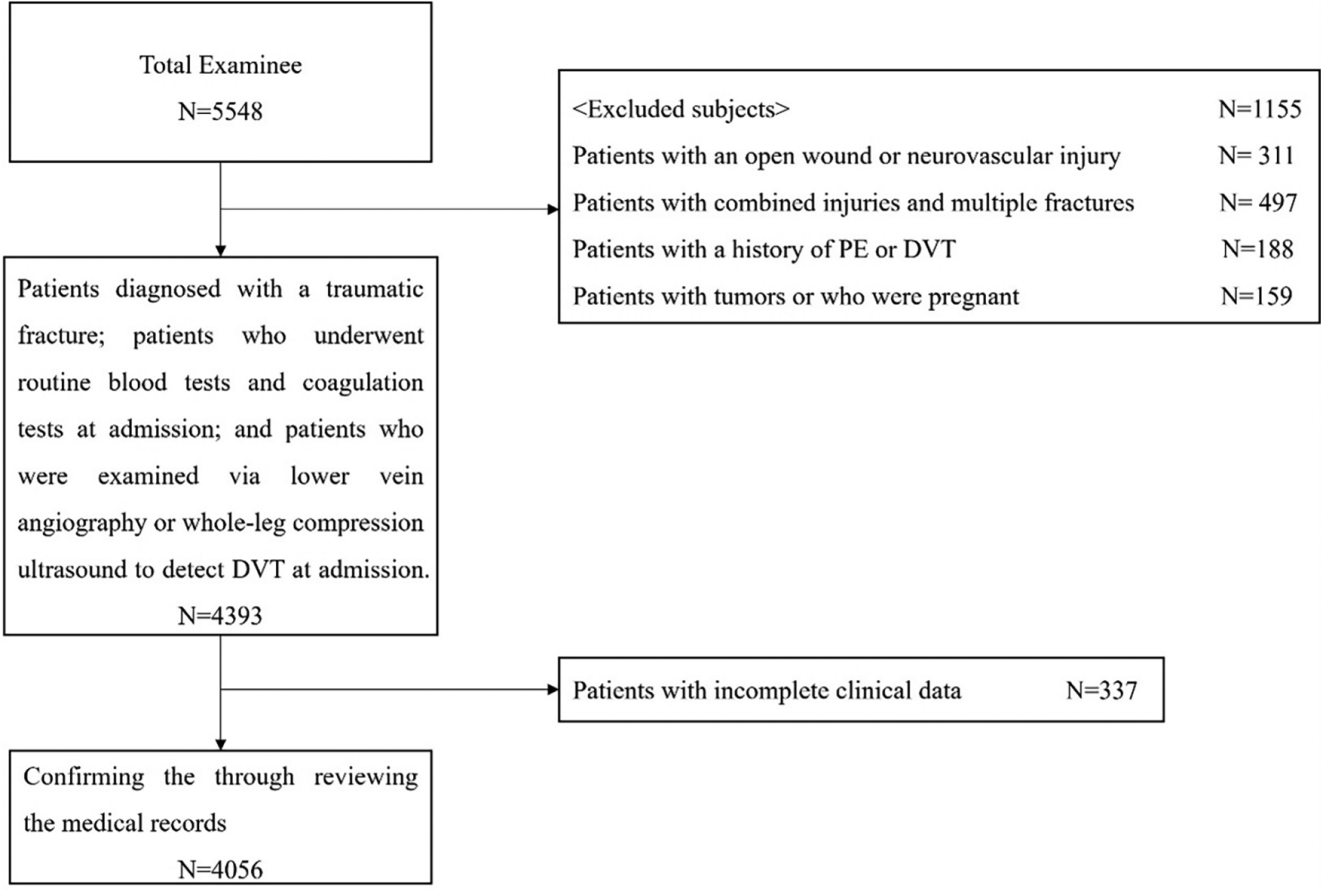
Process of selecting the study subjects.

This study was approved by our institutional review board. We reviewed the medical records of individuals with lower extremity and pelvic-acetabular fractures admitted to three hospitals in China from February 2018 to March 2023 and analyzed 4,056 patients who met the inclusion and exclusion criteria. The inclusion and exclusion criteria and the selection process are illustrated in Figure 1.

## Data collection

Patient data, including age, sex, height, weight, fracture site, injury mechanism, complications, smoking status, interval between injury and admission (h), results of routine blood tests and coagulation function tests at admission, and results of venography or ultrasonography of the lower extremities at admission, were collected. The fracture sites were classified as ankle-foot, tibia-fibula, peri-knee, femoral shaft, peri-hip, and pelvic-acetabular fractures. Injury mechanisms were classified as high- and low-energy injuries. The patients were classified as having proximal or non-proximal DVT based on venography or ultrasonography results of the lower extremities. Proximal DVT was defined as thrombosis involving popliteal vein and above, while non-proximal DVT was defined as isolated calf vein thrombosis (7).

## Statistical analysis

Statistical analyses were performed using SPSS (version 21.0; SPSS, Chicago, Illinois, USA). Measurement data are expressed as mean ± standard deviation (SD) and compared using two independent sample *t*-tests. Count data are reported as numbers (percentages) and were compared using the *χ*^2^ test. A multivariate logistic regression model was used to identify independent risk factors for proximal DVT. Moreover, the predictive value of the related risk factors for proximal DVT was further analyzed using a receiver operating characteristic (ROC) curve. The cutoff points of platelet count and D-dimer level were selected according to the maximum Youden index. Sensitivity, specificity, negative predictive value (NPV), and positive predictive value (PPV) for DVT diagnosis were also determined. Statistical significance was set at *P* < 0.05.

## Results

### Demographic characteristics of all patients

A total of 4,056 patients were evaluated, including 1,920 females (47.34%) and 2,136 males (52.66%) with a mean age of 54.32 years (SD, 19.46; range, 17–96 years). The mean body mass index (BMI) was 21.26 kg/m^2^ (SD, 3.58; range, 14.27–39.51 kg/m^2^). Of the 4,056 patients, 776 had ankle-foot fractures, 464 had tibia-fibula fractures, 616 had peri-knee fractures, 248 had femoral shaft fractures, 1,280 had peri-hip fractures, and 672 had pelvic-acetabular fractures. There were 3,383 cases and 673 cases of low- and high-energy injuries, respectively. The mean time from injury to admission was 23.65 h (SD, 19.66; range, 1.00–68.00 h).

### Prevalence of proximal DVT

**Figure 2:**
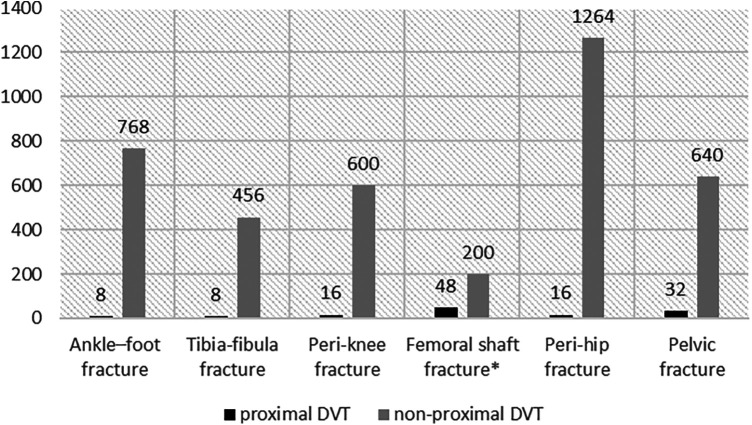
The distribution of DVT in different fracture sites. Femoral shaft fracture*: Patients with femoral shaft fractures had the highest prevalence at 19.35%.

The prevalence of proximal DVT was 3.16% (128/4,056). In patients with ankle-foot, tibia-fibula, peri-knee, femoral shaft, peri-hip, and pelvic-acetabular fractures, the prevalence rates of proximal DVT were 1.03% (8/776), 1.72% (8/464), 2.60% (16/616), 19.35% (48/248), 1.25% (16/1,280), and 4.76% (32/672), respectively (Figure 2). Except for pelvic fractures, proximal DVT was located in the injured lower extremities. Among patients with pelvic fractures, 77.27% (17/22) of proximal DVTs were located in the right lower extremity. PE was diagnosed based on computed tomography pulmonary angiography (CTPA) within 72 h of admission in 31 (24.22%) patients; all of them were proximal DVT cases (nine with femoral shaft fractures, 14 with femoral neck fractures, and eight with pelvic-acetabular fractures).

### Univariate analysis of risk factors

Males had a higher prevalence of proximal DVT than females (4.03% vs. 2.19%, *P* = 0.013). There were significant differences in BMI, fracture site, and interval between injury and admission between the two groups (*P* < 0.05). Patients with high-energy injuries had a higher incidence of proximal DVT than those with low-energy injuries (10.25% vs. 1.74%, *P* < 0.001). Patients with proximal DVT had a higher prevalence of diabetes (25.00% vs. 18.13%, *P* = 0.014) and a lower prevalence of coronary heart disease (CHD) (6.25% vs. 13.85%, *P* = 0.048) than those without proximal DVT. Patients with proximal DVT had a lower hematocrit level (35.62 ± 2.84% vs. 38.89 ± 6.31%, *P* = 0.031) and platelet count (156.87 ± 47.73 × 10^9^/L vs. 210.74 ± 75.75 × 10^9^/L, *P* = 0.005), and a higher D-dimer level (4.79 ± 4.86 mg/L vs. 2.91 ± 4.24 mg/L, *P* = 0.0141) (Table 1).

**Table 1:** Demographic characteristics and risk factors associated with admission proximal DVT.

Variables	Proximal DVT	Non-proximal DVT	Overall	*p*
Number	128 (3.16)	3,928	4,056	
Age (years)	52.37 ± 14.57	54.38 ± 19.60	54.32 ± 19.46	0.598
Sex				0.013*
Female	42 (32.81)	1,878 (47.81)	1,920 (47.34)	
Male	86 (67.19)	2,050 (52.19)	2,136 (52.66)	
BMI (kg/m^2^)				0.001*
18.5–23.9	32 (25.00)	2,000 (50.92)	2,032 (50.10)	
<18.5	8 (6.25)	328 (8.35)	336 (8.28)	
24.0–27.9	48 (37.50)	1,288 (32.79)	1,336 (32.94)	
≥28.0	40 (31.25)	312 (7.94)	352 (8.68)	
Fracture site				0.000*
Ankle–foot	8 (6.25)	768 (19.54)	776 (19.13)	
Tibia-fibula	8 (6.25)	456 (11.61)	464 (11.44)	
Peri-knee	16 (12.50)	600 (15.26)	616 (15.19)	
Femoral shaft	48 (37.50)	200 (5.09)	248 (6.11)	
Peri-hip	16 (12.50)	1,264 (32.17)	1,280 (31.56)	
Pelvic -acetabula	32 (25.00)	640 (16.33)	672 (16.57)	
Injury mechanism				0.000*
Low-energy injury^a^	59 (46.09)	3,324 (84.62)	3,383 (83.41)	
High-energy injury^b^	69 (53.91)	604 (15.38)	673 (16.59)	
Complication				
Hypertension	24 (18.75)	680 (17.31)	704 (17.36)	0.672
Diabetes	32 (25.00)	712 (18.13)	744 (18.34)	0.014*
Coronary heart disease^c^	8 (6.25)	544 (13.85)	552 (13.61)	0.048*
Stroke^d^	26 (20.31)	728 (18.53)	754 (18.59)	0.065
Smoking status	12 (9.38)	308 (7.84)	320 (7.89)	0.087
Interval between injury and admission (h)				0.000*
≤24	56 (43.75)	3,088 (78.62)	3,144 (77.52)	
24–48	16 (12.50)	440 (11.20)	456 (11.24)	
>48	56 (43.75)	400 (10.18)	456 (11.24)	
Serum markers at admission				
Hematocrit (%)	35.62 ± 2.84	38.89 ± 6.31	38.84 ± 6.29	0.031*
Hemoglobin (g/L)	127.00 ± 27.22	123.45 ± 21.71	123.56 ± 21.88	0.524
Platelets (×10^9^/L)	156.87 ± 47.73	210.74 ± 75.75	209.04 ± 75.59	0.005*
Prothrombin international ratio	1.04 ± 0.06	1.04 ± 0.09	1.04 ± 0.09	0.889
Partial thromboplastin time	27.12 ± 3.07	28.87 ± 4.40	28.81 ± 4.37	0.116
Thrombin time	16.86 ± 1.23	17.24 ± 1.90	17.23 ± 1.88	0.427
Prothrombin time	12.61 ± 0.75	12.58 ± 1.40	12.58 ± 1.38	0.945
Fibrinogen (g/L)	3.74 ± 1.39	3.56 ± 1.23	3.57 ± 1.23	0.574
D-dimer (mg/L)	4.79 ± 4.86	2.91 ± 4.24	2.97 ± 4.27	0.014*

DVT, deep vein thrombosis; BMI, body mass index.

^a^ Low-energy injury was defined as an injury which patients would sustain while falling over slippery ground in a walking or sitting position.

^b^ High-energy injury was defined as an injury where there was a high possibility that multiple organs might be damaged due to mechanisms such as falling more than 4 ft, traffic accident, and direct blow.

^c^ Coronary heart disease includes chronic myocardial ischemia, ST segment changes without clinical symptoms, and delayed myocardial infarction in electrocardiograms.

^d^ Stroke includes fresh and delayed cerebral ischemia and hemorrhage in computed tomography or magnetic resonance imaging.

* Significance at *p*-value < 0.05.

There were no significant differences in age, hypertension, stroke, smoking status, hemoglobin level, prothrombin international ratio, partial thromboplastin time, thrombin time, prothrombin time, or fibrinogen levels between the two groups (*P* > 0.05) (Table 1).

### Multivariate analysis of risk factors

BMI ≥ 24.0 kg/m^2^ (BMI: 24.0–27.9, odds ratio [OR] = 2.031, 95% confidence interval [CI] 1.030–4.595, *P* = 0.042; BMI: ≥28, OR = 6.788, 95% CI: 3.592–12.827, *P* = 0.001), femoral shaft fractures (OR = 20.848, 95% CI: 9.110–47.710, *P* = 0.011), high-energy injury (OR = 3.394, 95% CI: 2.078–7.976, *P* = 0.005), diabetes (OR = 3.583, 95% CI: 1.375–9.456, *P* = 0.010), and interval between injury and admission >24 h (interval between injury and admission: 24–48 h, OR = 3.104, 95% CI: 1.601–6.015, *P* = 0.001; interval between injury and admission: >48 h, OR = 20.530, 95% CI: 11.913–35.379, *P* < 0.001) were independent risk factors for proximal DVT. Patients with CHD (OR = 0.411, 95% CI: 0.238–0.709, *P* = 0.002) had a decreased risk of admission for proximal DVT (Table 2).

**Table 2:** Multivariate logistic regression analysis for risk factors associated with admission DVT.

Risk factors	COR (95%CI)	*P*	AOR (95% CI)	*P*
BMI (kg/m^2^)				
18.5–23.9	1.0 (reference)		1.0 (reference)	
<18.5	3.125 (0.554–7.625)	0.197	1.467 (0.822–2.618)	0.195
24.0–27.9	1.929 (1.510–4.290)	0.033*	2.031 (1.030–4.595)	0.042*
≥28.0	8.013 (2.062–31.138)	0.003*	6.788 (3.592–12.827)	0.001*
Fracture site				
Ankle–foot	1.0 (reference)		1.0 (reference)	
Tibia-fibula	1.684 (0.103–7.451)	0.714	1.048 (0.368–2.985)	0.975
Peri-knee	2.560 (0.228–8.772)	0.446	1.541 (0.626–3.794)	0.739
Femoral	20.040 (12.651–41.235)	0.004*	20.848 (9.110–47.710)	0.011*
Peri-hip	1.215 (0.109–3.582)	0.874	0.359 (0.020–6.303)	0.484
Pelvic-acetabular	4.800 (0.526–13.812)	0.164	2.029 (0.858–4.798)	0.569
Injury mechanism				
Low-energy injury	1.0 (reference)		1.0 (reference)	
High-energy injury	3.331 (4.007–5.217)	0.000*	3.394 (2.078–7.976)	0.005*
Diabetes	3.011 (2.773–7.221)	0.014*	3.583 (1.375–9.456)	0.010*
Coronary heart disease	0.541 (0.371–0.889)	0.001*	0.411 (0.238–0.709)	0.002*
Time from injury to admission				
≤24	1.0 (reference)		1.0 (reference)	
24–48	2.005 (1.406–9.899)	0.004*	3.104 (1.601–6.015)	0.001*
>48	7.720 (2.600–22.922)	0.000*	20.530 (11.913–35.379)	0.000*
PLT	0.986 (0.977–0.996)	0.004*	0.983 (0.970–0.996)	0.009*
D-dimer	1.065 (1.008–1.148)	0.008*	1.112 (1.001–1.235)	0.048*

COR, crude odds ratio; AOR, adjusted odds ratio; DVT, deep vein thrombosis; CI, confidence interval; BMI, body mass index; PLT, platelet.

* Significance at *p*-value < 0.05.

In addition, a lower platelet count (OR = 0.989, 95% CI: 0.970–0.996, *P* = 0.009) and high D-dimer levels (OR = 1.112, 95% CI: 1.001–1.235, *P* = 0.048) increased the risk of proximal DVT at admission (Table 2).

### ROC curve analysis for platelet and D-dimer value

**Figure 3:**
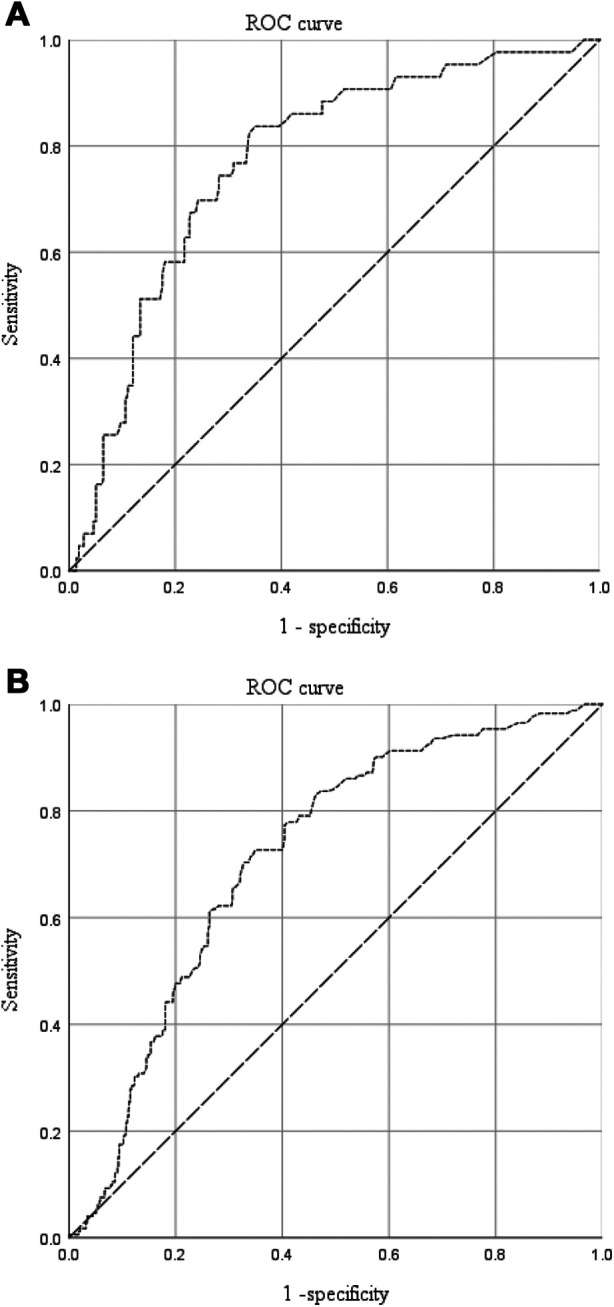
The predictive value of platelet counts (**A**) and D-dimer levels (**B**) for proximal DVT in patients with traumatic fractures.

ROC curve analysis was performed to determine the predictive values of platelet count and D-dimer level for proximal DVT (Figures 3A,B), and the detailed results are listed in Table 3. The area under the curve (AUC) was 0.721 for platelets and 0.704 for D-dimers. The cut-off points were 174 × 10^9^/L (sensitivity, 0.654; specificity, 0.750) and 2.18 mg/L (sensitivity, 0.813; specificity, 0.654) for platelet counts and D-dimer levels, respectively. The PPV and NPV were 0.078 and 0.985 for platelet counts and 0.071 and 0.990 for D-dimer level, respectively.

**Table 3:** Receiver-operating characteristic curve analysis of platelet and D-dimer.

Risk factors	Platelet	D-dimer
AUC	0.721	0.704
95% CI	0.610–0.832	0.574–0.834
*P*	0.003*	0.005*
Cut-off value	174 × 109/L	2.18 mg/L
Sensitivity	0.654	0.813
Specificity	0.750	0.654
PPV	0.078	0.071
NPV	0.985	0.990

AUC, area under the curve. CI, confidence interval. PPV, positive predictive value. NPV, negative predictive value.

* Significance at *p*-value < 0.05.

## Discussion

### Prevalence of proximal DVT

The prevalence of proximal DVT was 3.16%, similar to that reported by Hao et al. In their report, the incidence was 3.69% (59/1,596) (11). Of note, in this study, 24.22% of all patients with proximal DVT were diagnosed with PE within 72 h after admission. Girard et al. reported a higher incidence of PE in patients with proximal thrombosis (40%–50%) (12). Research suggests that the case fatality rate of PE is close to 50% (5). Therefore, for patients with proximal DVT, vital signs should be closely monitored, retrievable inferior vena cava filters should be implanted when necessary, and pulmonary angiography should be performed in patients with suspected PE to prevent fatal PE. Previous studies suggest that DVT is more likely to occur on the left side (13, 14). Due to the anatomical position, the left common iliac vein between the left common iliac artery and sacrum is prone to compression, which leads to slow blood flow in the left vein and thrombosis occurrence (15). However, we found that in patients with pelvic fractures, 77.27% of proximal DVT was located in the right lower extremity. This may be because in pelvic fractures, the right side of the body is the most commonly affected side; this results in the right vascular endothelium being more susceptible to injury (16). More importantly, Hou et al. found that patients with proximal acute lower extremity VTE were more likely to develop PE than those with distal VTE. Furthermore, patients with right-sided acute lower extremity VTE were at higher risk of symptomatic PE than were those with left-sided acute lower extremity VTE (17). Therefore, in patients with pelvic fracture, screening for right proximal DVT should be strengthened.

### Risk factors of admission DVT

Our study showed that age was not associated with proximal DVT occurrence, which is consistent with the findings of Nathan et al. They found that age was not a risk factor for proximal thrombosis (18). Studies have shown that female sex is an independent risk factor for DVT (19, 20). First, platelet reactivity is significantly higher in women than in men (21), and second, the common iliac vein (CIV) is more likely to be compressed in women (22, 23), leading to a higher incidence of DVT. This study showed that men had a higher incidence of proximal DVT. However, this was not an independent risk factor. It is speculated that this may be related to differences in trauma mechanisms between male and female patients. Regarding trauma, males are more likely to have high-energy fractures (24, 25). Previous studies have confirmed that high-energy injury is an independent risk factor for DVT (20, 26). In the present study, we obtained the same results.

Obesity is closely associated with the formation of DVT (27–29). Kornblith et al. (30) demonstrated that obese patients are more likely to have a hypercoagulable state after injury. Additionally, obese patients underwent less functional exercise and activity than non-obese patients, which increased the risk of abnormal venous valve pressure and hemodynamics (31). In our study, overweight and obesity were found to be independent risk factors for proximal DVT. The incidence of DVT is closely related to injury severity (32). Patients with overweight and obese tend to experience more severe injuries during the trauma process, which may be another reason why they are prone to proximal DVT. Ryb et al. (33) found that patients with (but not those with obesity) experienced more severe injuries, and Durgun et al. (34) found that the injury severity score (ISS) increased in proportion to increases in BMI.

The prevalence of DVT in trauma patients is related to the fracture sites. Additionally, Wang et al. found that femoral shaft fractures were associated with the highest incidence of proximal DVT (35). Yang et al. found that the incidence rates of proximal DVT at admission were as high as 14.81% (64/432) in patients with femoral shaft fractures (36). In our study, a higher incidence (19.35%) of proximal DVT was also found in patients with femoral shaft fractures; femoral shaft fractures were independent risk factors for proximal DVT.

Similar to most studies (37, 38), we found that diabetes was an independent risk factor for proximal DVT. Some studies have reported that patients with CHD are prone to DVT, which is related to the hypercoagulable state of the blood in patients with CHD (39, 40). However, we found that patients with CHD had a decreased risk of proximal DVT, this result may be attributed to long-term anticoagulation therapy. Platelets play an essential role in the pathogenesis of acute coronary syndromes, therefore an important part of the treatment of acute coronary syndromes, and of primary and secondary preventive measures in coronary heart disease, consists of antiplatelet treatment (41). Aspirin is now a commonly used antiplatelet agent in patients with coronary artery disease (42). Numerous studies have confirmed that aspirin significantly reduces the incidence of DVT (43–45). Bala et al. found that patients taking aspirin had the lowest incidence of deep vein thrombosis compared with those using other antiplatelet agents, including factor Xa inhibitors, enoxaparin, and warfarin (46).

A large number of studies have shown that the delay from injury to admission is an important factor leading to the high incidence of DVT in patients with lower extremity fractures (11, 20, 39). Our study showed that an interval between injury and admission of >24 h was an independent risk factor for proximal DVT in patients with lower extremity and pelvic-acetabular fractures. Hypercoagulability occurs 24 h after trauma (47), which may be the physiological basis of proximal DVT in patients with fractures. In addition, delayed anticoagulation owing to delayed hospital admission in patients with trauma may contribute to the development of proximal DVT. Wu et al. and Xia et al. found that delayed anticoagulation 24 h after trauma was positively correlated with the occurrence of VTE (48, 49).

Some previous studies have shown that patients with DVT have a significantly higher platelet count (50, 51). However, Sevuk et al. found that platelet counts were lower in patients with acute proximal DVT (52). A potential mechanism for low platelet count status in patients with proximal DVT is increased platelet consumption during the evolution of thrombosis (53). In the study, we found that platelet counts had a high NPV for proximal DVT formation in patients with traumatic fractures. In addition, the risk of proximal DVT was low in patients with a low clinical probability and a platelet count of not less than 174 × 10^9^/L. There is much accumulated evidence that DVT can be safely ruled out in patients with a low or intermediate clinical probability and a negative D dimer (<0.5 mg/L) without performing additional examinations. In this study, D-dimer was found to have a high NPV for proximal DVT. However, the cutoff value was as high as 2.18 mg/L. This was due to the fact that, except for venous thrombosis, elevated levels of D-dimer are also found in patients in whom coagulation and fibrinolysis are co-activated, such as those with recent trauma or surgery and those with severe sepsis (54).

Therefore, in patients with traumatic fractures, special attention should be paid to those with BMI ≥ 24.0 kg/m^2^, femoral shaft fractures, high-energy injury, diabetes and interval between injury and admission >24 h, and early prophylaxis and treatment plans should be formulated to prevent proximal thrombosis extension and acute PE. During thrombosis screening, DVT could be safely ruled out in patients with a low clinical probability and a platelet count >174 × 10^9^/L or D-dimer level <2.18 mg/L without performing additional examinations.

### Limitations of this study

This study has four limitations. First, the retrospective design has its inherent limitation of accuracy in data collection. Second, the diagnostic value of duplex ultrasonography for DVT remains controversial; most patients in this study only underwent ultrasound examination without venography, which may have led to underdiagnosis. Although venography is the gold standard for diagnosing lower extremity thrombosis, it is an invasive procedure that requires a specific work scenario. Therefore, venography is not routinely used to screen for lower extremity thrombosis. In addition, Cavaye et al. found that duplex scanning produced sufficiently accurate data on the diagnosis of lower limb DVT to warrant its clinical use (55). Canakci et al. found that point-of-care ultrasound had high specificity and sensitivity for the examination of the popliteal and femoral veins by an emergency physician to evaluate patients with a preliminary diagnosis of DVT (56). Barrellier et al. found that the prevalence of duplex-ultrasonography-detected venous thrombosis in patients with suspected or proven PE was equivalent to the rates reported in phlebography and autopsy series (57). Third, we excluded patients with combined injuries and multiple fractures, which might limit wider application of the findings. Wu et al. (49) and Shi et al. (58) found that patients with multiple trauma had a higher risk of DVT. Additionally, Song et al. (59) found that combined cranial trauma was an independent risk factor for preoperative DVT. Therefore, more attention should be paid to patients with combined injuries or multiple fractures. Fourth, this study only identified the associated risk factors for proximal DVT in patients with lower extremity and pelvic-acetabular fractures. However, external validation studies were still lacking, which was a drawback of this study.

## Conclusions

The prevalence of proximal DVT upon admission in patients with lower extremity and pelvic-acetabular fractures was 3.16%. BMI ≥ 24.0 kg/m^2^, femoral shaft fractures, high-energy injury, diabetes, and interval between injury and admission >24 h were independent risk factors for proximal DVT. However, the presence of CHD decreased the risk of proximal DVT. Platelet counts and D-dimer levels were effective indicators for excluding proximal DVT occurrence, with cut-off values of 174 × 10^9^/L and 2.18 mg/L, respectively. These epidemiologic data are helpful in the assessment and risk stratification of admission proximal DVT, and supporting the formulation of an early prophylaxis and treatment plan for DVT.

## Data Availability

The raw data supporting the conclusions of this article will be made available by the authors, without undue reservation.
